# ERRα as a Bridge Between Transcription and Function: Role in Liver Metabolism and Disease

**DOI:** 10.3389/fendo.2019.00206

**Published:** 2019-04-05

**Authors:** Hui Xia, Catherine R. Dufour, Vincent Giguère

**Affiliations:** ^1^Goodman Cancer Research Centre, McGill University, Montréal, QC, Canada; ^2^Department of Biochemistry, McGill University, Montréal, QC, Canada; ^3^Medicine and Oncology, McGill University, Montréal, QC, Canada

**Keywords:** nuclear receptor, metabolism, high-fat diet, diabetes, non-alcoholic fatty liver disease, inflammation, liver cancer

## Abstract

As transcriptional factors, nuclear receptors (NRs) function as major regulators of gene expression. In particular, dysregulation of NR activity has been shown to significantly alter metabolic homeostasis in various contexts leading to metabolic disorders and cancers. The orphan estrogen-related receptor (ERR) subfamily of NRs, comprised of ERRα, ERRβ, and ERRγ, for which a natural ligand has yet to be identified, are known as central regulators of energy metabolism. If AMP-activated protein kinase (AMPK) and mechanistic target of rapamycin (mTOR) can be viewed as sensors of the metabolic needs of a cell and responding acutely via post-translational control of proteins, then the ERRs can be regarded as downstream effectors of metabolism via transcriptional regulation of genes for a long-term and sustained adaptive response. In this review, we will focus on recent findings centered on the transcriptional roles played by ERRα in hepatocytes. Modulation of ERRα activity in both *in vitro* and *in vivo* models via genetic or pharmacological manipulation coupled with chromatin-immunoprecipitation (ChIP)-on-chip and ChIP-sequencing (ChIP-seq) studies have been fundamental in delineating the direct roles of ERRα in the control of hepatic gene expression. These studies have identified crucial roles for ERRα in lipid and carbohydrate metabolism as well as in mitochondrial function under both physiological and pathological conditions. The regulation of ERRα expression and activity via ligand-independent modes of action including coregulator binding, post-translational modifications (PTMs) and control of protein stability will be discussed in the context that may serve as valuable tools to modulate ERRα function as new therapeutic avenues for the treatment of hepatic metabolic dysfunction and related diseases.

## Introduction

The concept of direct transduction of simple chemical changes into distinct physiological effects was definitively established by the elucidation of the mechanisms of action of nuclear receptors (NRs), which interact with the genome and directly regulate gene transcription in response to chemical ligands like lipophilic hormones, vitamins, various metabolites, and synthetic drugs (1, 2). The discovery of this ligand-dependent response system ignited a new era in molecular endocrinology. Of the 48 human NRs, there are a few for which appropriate endogenous ligands have yet to be identified and thus are termed orphan receptors (3, 4). Estrogen-related receptors (ERRs) were the first orphan NRs identified and this sub-family of NRs now consist of ERRα, ERRβ, and ERRγ (5, 6). Although ERRs do share sequence similarities with estrogen receptors (ERs), they are not activated by estrogens, making the name “estrogen-related receptors” inappropriate or unfortunately even misleading. As the ERRs have been established as major regulators of energy metabolism (7), a more suitable acronym for ERR would be “energy-related receptors.” While many orphan NRs were eventually “de-orphanized,” the ERRs remain to this date orphans (4). In this review, our focus will be mainly on the most-studied member of the ERR subfamily, ERRα, specifically in the transcriptional control of hepatocellular functions as significant efforts in the last decade have been made toward unraveling the role of this receptor in liver health and disease.

Given the lack of ligands to directly regulate ERRα activity, initial study of the transcriptome and cellular pathways regulated by ERRα was hampered as compared with other NRs. However, integration of gene expression profiles and genome-wide chromatin immunoprecipitation (ChIP)-based studies together with in-depth phenotypic analyses of ERRα-null mice then facilitated the studies of ERRα cellular functions and rapidly advanced our understanding and appreciation of ERRα as a major transcriptional regulator of mitochondrial function and metabolism. While reviews discussed the primary functions of ERRα in diverse contexts (8–11), its pivotal role in liver homeostasis deserves an independent review. The liver is an essential metabolic organ that governs whole-body energy metabolism and dysregulation of hepatic homeostasis is a major contributor to the metabolic syndrome including insulin resistance, non-alcoholic fatty liver disease (NAFLD) and type 2 diabetes. It has been shown that liver energy metabolism is under strict regulation by numerous NRs as well as their coregulators, whose activities are regulated by upstream signals like insulin, glucagon as well as other metabolic hormones (12, 13). Herein, we will provide a brief overview of ERRα, its role as a sensor of intrinsic and environmental cues, highlight ERRα-driven hepatic transcriptional gene networks that underscore its key role in energy homeostasis and discuss the potential benefits of modulating ERRα activity to prevent and/or treat liver metabolic dysfunction and diseases.

## The Orphan Nuclear Receptor ERRα

### Structure and Function

The three ERRs comprise the NR3B subgroup, which belongs to the larger NR3 subfamily of classic steroid receptors, including the ERs, androgen, progesterone, mineralocorticoid, and glucocorticoid receptors (14, 15). ERRα (NR3B1, *Esrra*) was initially discovered in 1988 in a screen designed to identify novel steroid hormone receptors closely related to human ERα and consequently named ERRα (5). However, it turned out that this new receptor did not bind natural estrogens, or other known steroid hormones as well as their derivatives, and as such it was recognized as the first orphan nuclear receptor. ERRβ (NR3B2, *Esrrb*) was identified by using ERRα cDNA as a probe (5) while ERRγ (NR3B3, *Esrrg*) was discovered a decade later (16). The expression of ERRα is ubiquitous and is elevated in metabolically active tissues such as the heart, kidneys, intestine, skeletal muscle, brown adipose tissue (BAT) and liver. Generally, the expression of ERRα is more abundant than the other two ERR members (7, 10).

ERRα possesses the characteristic structural features typical to NRs, including a non-conserved amino-terminal domain (NTD), a central zinc finger DNA-binding domain (DBD), and a functional C-terminal ligand-binding domain (LBD) (15). Notably, the three ERRs share considerable structural relatedness in their NTDs, which is typically poorly conserved among NRs. For example, ERRα and ERRγ both contain a functional phospho-sumoyl switch motif in this domain (17, 18), indicating an important role of the NTD in the regulation of ERR transcriptional activities. The ERRs regulate gene expression via binding to a specific DNA sequence in regulatory regions located in the promoter or at a distal site from the transcriptional start site of a target gene, referred to as the ERR element (ERRE). By using an unbiased binding site selection approach, the binding motif for the ERRs was defined as TCAAGGTCA (19). This motif was validated to serve as the main ERR binding site *in vivo* by bioinformatics analysis of a large set of ERR target promoters identified in different cell types through ChIP coupled with genomic DNA array technology (ChIP-on-chip) (20–22) and later confirmed by ChIP-seq studies (23–26). The DBD sequence of ERRα is highly conserved with that of ERRβ and γ, therefore most if not all ERR target genes can be targeted by all three ERR isoforms (20, 26). Indeed, the ERRs have the ability to bind to the ERRE not only as a monomer or homodimer but also as heterodimer composed of two distinct ERR isoforms (20, 27–29). The LBD of the ERRs contain a well-conserved activation function-2 (AF-2 helix) motif that is positioned in the active configuration even in the absence of a ligand (30, 31). Thus, the ERRs display significant constitutive transcriptional activity that is dependent on the interaction with coregulators, which are often considered as protein ligands for the ERRs (32).

### Regulation of ERRα Activity by Transcriptional Co-regulators

ERRα activates or represses gene expression in response to different cellular signals, being highly dependent on the presence of its co-regulators in specific tissues or cultured cell lines. The peroxisome proliferator-activated receptor γ (PPARγ)-coactivator 1 α (PGC-1α) is the most notable and potent coactivator of ERRα (33–37). PGC-1α has been shown to play an essential role in mitochondrial biogenesis, oxidative phosphorylation (OXPHOS), fatty acid β-oxidation (FAO), adaptive thermogenesis, glucose uptake, glycolysis, hepatic gluconeogenesis, ketogenesis, and circadian activity via selectively interacting with and co-activating diverse transcription factors (38, 39). PGC-1α and ERRα display similar expression patterns, being expressed in tissues reliant on oxidative metabolism for elevated energy requirements, such as the heart, skeletal muscle, BAT and liver (19, 40, 41). Indeed, PGC-1α, PGC-1β, and ERRα have shown a functional co-dependency in the control of vast metabolic gene networks in numerous tissues (24, 42–46). The ERRα/PGC-1α functional complexes were first identified in a yeast two-hybrid screen of a cardiac cDNA library (33). Prior to this discovery, *Acadm* was identified as the first *bona fide* ERRα target gene, encoding medium-chain acyl coenzyme A (MCAD), which catalyzes the initial step in mitochondrial FAO (19, 40). Moreover, ERRα binds to a distal enhancer of *Ppargc1a* to drive its expression (47). In turn, expression of PGC-1α coactivates ERRα, forming a feed-forward loop to promote the expression of metabolic genes (34, 36, 47, 48). Interestingly, *in vitro* binding experiments demonstrated that ERRα binds PGC-1α via a leucine-rich motif which is specifically recognized by the ERRs (33, 49). The utilization by the ERRs of a distinct PGC-1α interaction interface offers the opportunity to regulate ERR/PGC-1α signaling via post-translational modifications (PTMs) of either partner.

The NR corepressor 1 (NCoR1) is a well-characterized and ubiquitously expressed corepressor. It regulates gene transcription by forming a large protein complex in which the chromatin modifying enzyme histone deacetylase 3 (HDAC3) is a core component (50). Current studies propose a yin-yang relationship between PGC-1α and NCoR1 that confers opposing effects on the transcriptional activity of ERRα (51, 52). Indeed, global gene expression analysis revealed a high overlap between the effects of PGC-1α overexpression and NCoR1 deletion on metabolic genes in muscle, and consistent with this, the stimulatory effect of PGC-1α on OXPHOS gene expression specifically counteracts NCoR1-mediated repression of ERRα. The use of a common binding pocket by different coactivators and corepressors suggests a critical regulation of this cofactor exchange (53). NCoR1 is a basal corepressor, thus it seems to repress ERRα under basal conditions and is exchanged with coactivators upon physiological stimuli such as cold exposure and exercise (51). The homeodomain-containing protein PROX1 can also form an inhibitory complex with ERRα and PGC-1α (22). PROX1 was shown to occupy the promoters of metabolic genes on a genome-wide scale and bind to ~40% of ERRα target genes (22). Furthermore, ERRα transcriptional activity can atypically be repressed or activated by the NR interacting protein 140 (RIP140) (54–56). RIP140 has been shown to repress several genes involved in glucose and lipid metabolism. Also, RIP140 expression is up-regulated by ERRα during adipogenesis suggesting a role for the RIP140/ERRα complex in maintaining energy homeostasis in adipocytes (57–59). In summary, the shift between different ERRα-regulated pathways in response to physiological and metabolic cues is likely facilitated through interactions with distinct coregulators.

### Regulation of ERRα Activity by Post-transcriptional and Post-translational Control Mechanisms

The activity of ERRα is dynamically modulated post-transcriptionally by microRNAs (miRNAs). miRNAs are endogenous small non-coding RNAs of ~22 nucleotides in length, which have recently emerged as important regulators of gene expression in many diseases by targeting messenger RNAs (mRNAs) for degradation or translational repression (60, 61). *ESRRA* is a direct target of miRNA-137 and miR-125a (62–65). miRNA-137-mediated *ESRRA* mRNA degradation contributes to the impaired proliferative and migratory capacity of breast cancer (BCa) cells as well as that of placenta trophoblast cells through reduced expression of the ERRα-regulated gene *WNT11*. miR-125a, by targeting *ESRRA* mRNA, reduces the proliferation and invasion of oral squamous cell carcinoma cells. miR-125a also negatively regulates porcine pre-adipocyte differentiation, partly via suppressing ERRα action. The activity of ERRα is also regulated by PTMs including ubiquitination, phosphorylation, sumoylation and acetylation. The protein level of ERRα is under the control of the ubiquitin-proteasome system (UPS). Parkin, an E3-ubiquitin (Ub) ligase whose mutations cause Parkinson's disease, reduces dopamine toxicity and oxidative stress by promoting ERRα ubiquitination and degradation, and thus abolishing ERRα-mediated activation of monoamine oxidase (MAO) promoters (66). Furthermore, mTOR was shown to positively regulate ERRα protein stability and activity via transcriptional control of the UPS involving repression of the genes *Stub1* and *Ubb* (23). ERRα is also a phosphoprotein that is phosphorylated on multiple sites. Barry et al. first reported that epidermal growth factor (EGF) treatment of MCF-7 cells enhanced ERRα phosphorylation, DNA binding, homodimerization, interaction with PGC-1α and transcriptional activity by activating protein kinase Cδ (PKCδ) (67). Ariazi et al. subsequently found that ERRα was phosphorylated *in vitro* by MAPK and AKT proteins, downstream kinases of the EGFR/ErbB2 (HER-2) signaling pathway, and that ErbB2 signaling elevated ERRα phosphorylation levels and transcriptional activity in BCa (68). Furthermore, cAMP has been shown to increase ERRα phosphorylation and nuclear localization either via the cAMP-PKA signaling cascade in lung type II cells (69) or by the cAMP-PI3K-ERK signaling pathway in prostate stromal cells (70). Serine residues 19 and 22 of ERRα serve as the major sites of phosphorylation in BCa cells and phosphorylation at serine 19 represses ERRα transcriptional activity via its effects on sumoylation of ERRα on lysine 14 (17, 18). Moreover, the affinity of ERRα for binding to ERREs is affected by a dynamic acetylation/deacetylation switch of four highly-conserved lysine residues within the DBD mediated by the acetyltransferase p300 coactivator associated factor (PCAF) and deacetylases, HDAC8 and sirtuin 1 homolog (SIRT1) (71). It must also be noted that ERRα activity can also be influenced by PTMs of its coregulators. For example, insulin induces the phosphorylation of NCoR1 on serine 1460 via AKT activation and this PTM selectively favors the interaction between NCoR1 and ERRα, thus repressing ERRα target genes involved in oxidative metabolism and liver fatty acid catabolism (72). Another example is HDAC3, which usually acts as a transcriptional corepressor together with NCoR1. However, HDAC3 activates ERRα in BAT by de-acetylating PGC1α, promoting the transcription of *Ucp1* and OXPHOS genes to ensure survival upon exposure to prolonged cold exposure (47). Together, microRNA targeting of *ESRRA* mRNA and PTMs of ERRα and its coregulators (summarized in Table 1) demonstrate that the ligand-independent transcriptional activity of ERRα can be dynamically and tightly regulated in response to changing metabolic signals.

**Table 1:** Known regulators of ERRα transcriptional activity.

**Factor**	**Mechanism**	**Effect on ERRα activity**	**References**
PGC-1α	Coactivator	Increases	(33–35, 37)
PGC-1β	Coactivator	Increases	(32, 42, 46, 73)
SRC	Coactivator	Increases	(74, 75)
PNRC2	Coactivator	Increases	(76)
NCoR1	Corepressor	Represses	(51, 52, 72)
PROX1	Corepressor	Represses	(22)
RIP140	Corepressor/coactivator	Represses/increases	(54–56)
miR-137	Targets 3′UTR of *ESRRA*	Represses	(62, 63)
miR-125a	Targets 3′UTR of *ESRRA*	Represses	(64, 65)
Parkin	Ubiquitination	Represses	(66)
mTOR	UPS repression	Increases	(23)
PCAF	Acetylation	Represses	(71)
HDAC8	Deacetylation	Enhances ERRα DNA binding affinity	(71)
SIRT1	Deacetylation	Enhances ERRα DNA binding affinity	(71)
PIASy	Sumoylation (Lys^14^ and Lys^403^)	Represses	(17, 18)
Kinase	Phosphorylation (Ser^19^)	Represses	(17, 18)
PKCδ	Phosphorylation	Enhances ERRα DNA binding affinity	(67)
MAPK	Phosphorylation	Increases	(68)
AKT	Phosphorylation	Increases	(68)
PKA	Phosphorylation and nuclear translocation	Increases	(69)
PI3K-ERK	Phosphorylation and nuclear translocation	Increases	(70)
HDAC3	Deacetylation of PGC1α	Increases	(47)

## Role of ERRα in Adaptation to Energy Demands and Environmental Cues

Dynamic regulation of gene networks by ERRα is required for the bioenergetic and functional adaptation to environmental stresses. External stimuli such as cold exposure upregulates the expression of ERRα as well as its coactivator PGC-1α in BAT and skeletal muscle of mice (35, 47), promoting thermogenesis through mitochondrial OXPHOS to adapt to cold environments. ERRα-null mice are unable to maintain body temperature in response to cold exposure because of a failure of mitochondrial biogenesis and oxidative capacity (77, 78). ERRα expression is also stimulated by exercise in a pattern parallel to that of PGC-1α in animal models and in humans (79–81). Accordingly, ERRα-null mice are hypoactive and exercise intolerant because of a reduced basal metabolic oxidative capacity (82–84). Moreover, the molecular clock serves as another input signal modulating ERRα levels in a circadian manner in tissues including liver and WAT (84–87). Furthermore, ERRα adapts to nutritional challenges such as increased intake of a lipid-rich diet or cycles of nutrient deprivation and availability by modulating metabolic gene and metabolite levels (88, 89). Rapamycin treatment, which is known to mimic amino-acid-like starvation, as well as glucose and amino acids deprivation also affect ERRα protein stability and transcriptional networks (23).

## ERRα-Dependent Transcriptional Networks

Given the lack of a natural ligand or high affinity pharmacological agent that can activate ERRα, identification of biological pathways modulated by ERRα is usually achieved by overexpression of PGC-1α or ERRα expression itself followed by gene expression studies and bioinformatics analyses. Genetic deletion or ERRα knock-down experiments have also been applied to elucidate ERRα-mediated changes in transcriptomes. One common problem of these perturbation assays is that the identification of gene expression profiles cannot easily differentiate between primary and secondary targets of ERRα. The initial characterization of direct ERRα target genes was based on identifying ERRα binding sites through manual inspection and functional analyses of promoter regions of ERRα-responsive genes. Although inefficient, integration of the above approaches helped to uncover multiple ERRα target genes and implicated ERRα in the regulation of FAO (19, 40), gluconeogenesis (90), lipid transport and uptake (91), as well as mitochondrial biogenesis and function (34, 92, 93). These ERRα targets were later validated by a series of genome-wide ChIP-based studies generated from the work of our laboratory (20–23, 25, 42, 83). ChIP-qPCR, ChIP-on-chip and ChIP-seq techniques have been improved and developed during the last decade, enabling high-confidence large-scale and genome-wide location analyses of NRs for target gene discovery (94). The characterization of comprehensive ERRα transcriptional gene networks stemmed from the work published by Dufour et al. with the first report of a genome-wide study of ERRα performed using ChIP-on-chip analyses on mouse heart (20). In the study by Charest-Marcotte et al., the use of ChIP-on-chip experiments on mouse liver led to the discovery of a genomic and functional relationship between ERRα and Prox1 (22). An important finding of this study was the discovery of the ERRα bioenergetic regulon, a cluster of functionally linked genes involved in the generation of energy from glucose. ERRα was found recruited to genes encoding virtually all enzymes involved in glycolysis, pyruvate metabolism, and the tricarboxylic acid (TCA) cycle aside the previously confirmed OXPHOS genes. Furthermore, by performing a comparative analysis of genome-wide binding of nuclear mTOR and ERRα by ChIP-seq, Chaveroux et al. revealed that mTOR is recruited to pol-III-transcribed genes to control mRNA translation and also to a large subset of pol-II-driven gene programs involved in UPS, insulin signaling, OXPHOS and fatty acid metabolism (23). Although this study showed that nuclear mTOR and ERRα co-binding to genomic loci were rare events, the two factors were found to co-regulate numerous genes implicated in the transcriptional regulation of common metabolic processes such as the TCA cycle and lipogenesis.

## ERRα Gene Networks in the Liver

**Figure 1:**
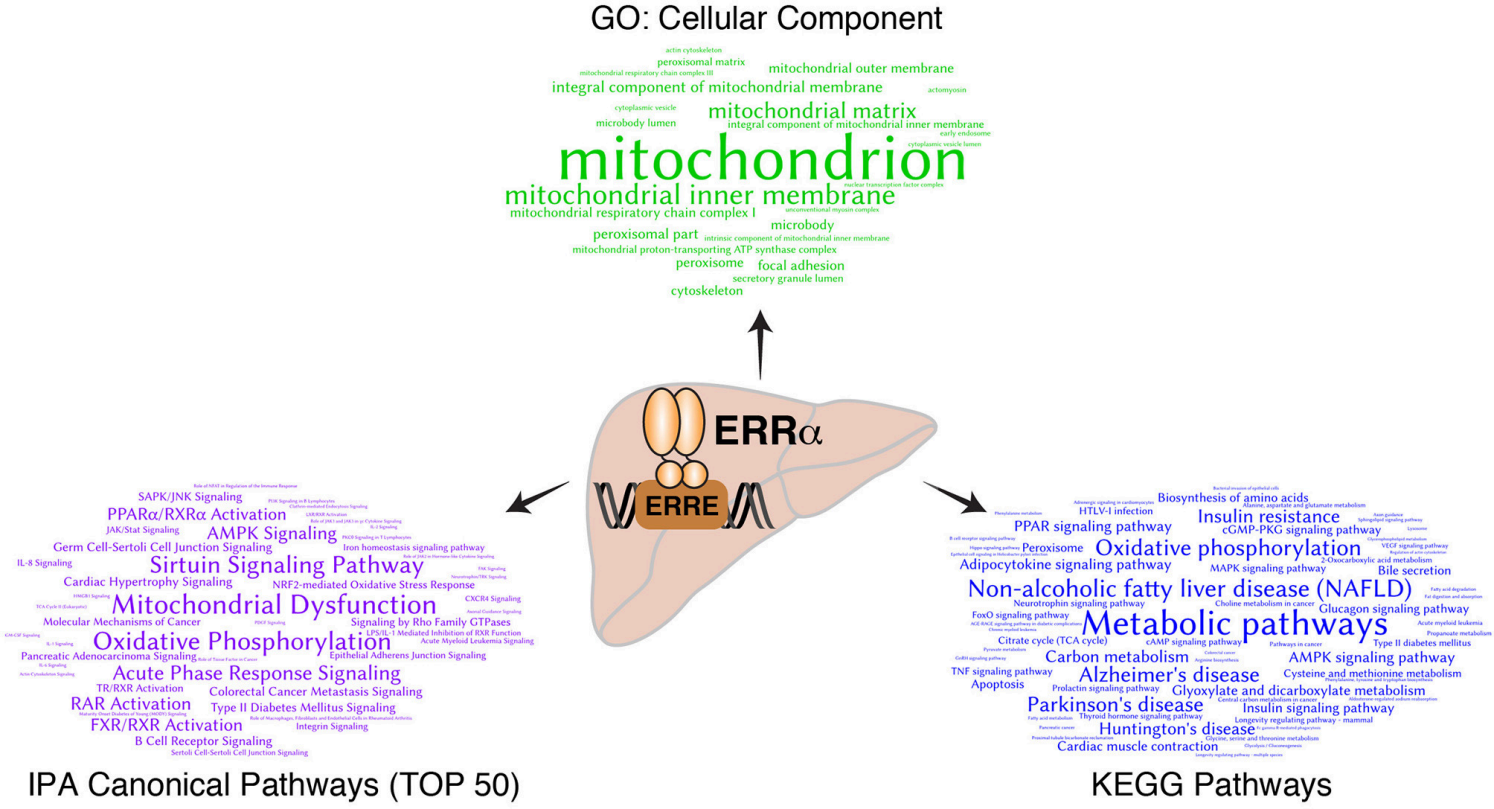
Bioinformatics analysis of direct ERRα target genes in mouse liver. Word clouds representing significantly overrepresented GO cellular component terms (top, green), KEGG pathways (right, blue) and the top 50 canonical pathways from Ingenuity Pathway Analysis (IPA) (left, purple) from the list of ERRα target genes with DNA binding events observed within ±10 kb of their transcription start sites as identified by ChIP-seq analysis (23). The size of the significant terms is reflective of their associated *p*-values whereby the most significant terms having lower *p*-values are displayed in larger font size.

**Figure 2:**
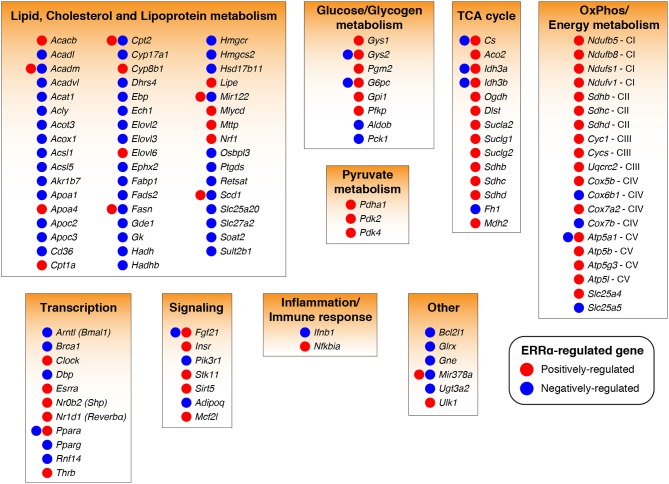
ERRα-regulated target genes and programs in liver cells. ERRα target genes (104) found transcriptionally regulated by ERRα in a positive (increased by ERRα, red) or negative (decreased by ERRα, blue) manner in hepatocytes from *in vivo* and *in vitro* studies involving genetic or pharmacological manipulation of ERRα activity are shown. Only ERRα-regulated genes having a high confidence for direct gene regulation by ERRα as determined by the presence of an ERRα-binding event within ±20 kb of the gene transcriptional start site were considered (22, 23). The genes, *Sdhb, Sdhc*, and *Sdhd* were associated to two biological programs. See also Table S1 for more information.

Bioinformatics analysis of ERRα target genes identified from the mouse liver ChIP-seq analysis (23) corroborated the early finding that ERRα is a regulator of mitochondrial function and metabolism (Figure 1). Word clouds illustrating the functional analysis of ERRα target genes using Gene Ontology (GO) cellular component, Kyoto Encyclopedia of Genes and Genomes (KEGG), and Ingenuity Pathway Analysis (IPA) are shown with lower *p*-value associated terms displayed in larger font size. Genes targeted by ERRα are more significantly enriched for terms related to mitochondria and energy metabolism (e.g., mitochondrial dysfunction, OXPHOS) as well as diseases relating to metabolic dysfunction including NAFLD, insulin resistance, Alzheimer's and Parkinson's disease. Genetic or pharmacological manipulation of ERRα in rodent and cell-based studies have further characterized the genes and biological programs regulated by ERRα, establishing a major role of ERRα in liver homeostasis. A list of currently known genes found either activated or repressed by ERRα in hepatocytes in a context-specific manner with evidence for direct binding of ERRα within ± 20 kb of the TSS from ChIP-based studies is summarized in Table S1 (22, 23, 82, 84, 88, 90, 95–103). A schematic of these direct ERRα-regulated genes associated to general biological processes are shown in Figure 2. Notably, genes related to lipid metabolism are found mostly repressed by ERRα in stark contrast to genes associated with mitochondrial energy production found largely positively-regulated by ERRα.

### ERRα Transcriptional Regulation of Glucose Metabolism

The liver is a key organ in regulating glucose homeostasis especially during periods of fasting and refeeding. Several genes with established roles in glucose handling have been shown to be influenced by ERRα. For example, transcription of *PCK1* which encodes the rate-determining enzyme phosphoenolpyruvate carboxykinase (PEPCK) in gluconeogenesis is repressed by ERRα in hepatocytes (90, 105). Interestingly, ERRα represses gluconeogenesis by antagonizing the stimulatory effect of PGC-1α, possibly via inhibiting the recruitment of PGC-1α to the proximal regulatory region of *PCK1* (90). Indeed, expression of *Pck1* is significantly up-regulated in ERRα-null liver during the light phase of the circadian cycle at which time gluconeogenesis is active (84). However, no difference in the levels of *Pck1* mRNA between fasted wild-type and ERRα-null mice were observed (90). The expression of *PCK1* is under intense hormonal regulation and is induced during periods of fasting to maintain circulating glucose levels, which is inconsistent with the observation that hepatic expression of ERRα is also upregulated by fasting in normal mice (105, 106). The physiological role of ERRα in liver is to repress gluconeogenesis under fed conditions. Additionally, ERRα activation by PGC-1α induces the transcription of *Gck* encoding glucokinase (Gck) which phosphorylates glucose and participates in glucose utilization by stimulating glycolysis and glycogen synthesis in liver. *Gck* is also induced by insulin, which is partly mediated by ERRα (107, 108). Absence of ERRα in HepG2 cells impaired the reliance on glycolysis in the presence of inhibitors of mitochondrial function (22). These studies suggest that enhancing the transcriptional activity of ERRα in the fed state might have beneficial effects on glucose metabolism through suppression of hepatic gluconeogenesis as well as simultaneous activation of glycolysis and glycogen synthesis. Unexpectedly, despite the increased expression of gluconeogenic genes in the liver of fed ERRα null mice, blood glucose levels were normal in the fed state (84, 90), which might result from increased glucose oxidation in the absence of ERRα. Indeed, ERRα has been shown to inhibit glucose oxidation by transcriptionally activating *PDK4*, which encodes pyruvate dehydrogenase kinase 4 (PDK4), in muscle and hepatocytes (37, 103, 109). PDK4 inhibits cellular glucose utilization by phosphorylating and inactivating the pyruvate dehydrogenase complex (PDC), which allows pyruvate entry into the TCA cycle (12). Therefore, ERRα, via up-regulating the level of PDK4, supports a switch from glucose oxidation to FAO and ultimately leads to reduced glucose metabolism (37, 103, 109). Overall, ERRα seems to play multiple and contradictory roles in glucose metabolism.

### ERRα Transcriptional Regulation of Lipid Metabolism

ERRα has be shown to play a fundamental role in lipid homeostasis. It is highly expressed in tissues that derive energy from fatty acid metabolism, likely contributing to the high basal levels of fatty acid utilization genes in these oxidative tissues (110, 111). Indeed, ERRα and MCAD are co-expressed in tissues with high energy needs. MCAD, whose expression levels are tightly regulated by tissue energy demands and dictate the rate of tissue FAO, catalyzes the first step in the mitochondrial oxidation of fatty acids (19, 40, 77, 112). Therefore, the initial finding that ERRα promotes the transcription of the MCAD gene (*Acadm*) (19, 40) and the further confirmation of the direct recruitment of ERRα at the *Acadm* promoter *in vivo* (20, 77) strongly suggests that ERRα activity increases fatty acid oxidation rates. However, gene expression profiling of adipose tissue from ERRα-null mice revealed an up-regulation of *Acadm* expression (113), whereas, analysis of adipose and muscle tissues from ERRα KO mice fed a high-fat diet (HFD) revealed no changes in the expression of this gene (100), suggesting that ERRα regulation of *Acadm* is nutrient-dependent. Genetic or pharmacological inhibition of ERRα leads to decreased lipid accumulation, reduced fat mass and resistance to HFD-induced obesity, partly because ERRα-null mice have a lower capacity for lipid absorption by the intestine (84, 91, 100, 113, 114). The intestine markedly contributes to total body FAO since it is essential for the uptake and transport of dietary fat, the first step in the energy chain (91). Microarray studies demonstrated that in addition to several down-regulated OXPHOS genes, the expression levels of a set of genes encoding proteins involved in lipid digestion and absorption were also altered in the ERRα-deficient intestine, including apolipoprotein (apo)A-IV (91). Furthermore, ERRα can stimulate adipogenesis via enhancing triglyceride (TG) accumulation and elevating expression of genes involved in lipid and energy metabolism in white adipose tissue (WAT), such as *Fasn*, the gene encoding fatty acid synthase (115, 116). Accordingly, ERRα-null mice display significantly decreased lipogenesis in WAT consistent with their leanness and decreased body weight gain in comparison to littermate controls chronically fed a HFD. The beneficial effects for loss of ERRα function in protection from HFD-induced body weight gain resulted also from a nearly 2-fold reduction in *de novo* hepatic lipogenesis (88). Although systemic ablation of ERRα protects mice from HFD-induced NAFLD, the presence of ERRα promotes the reversal of fasting-induced NAFLD by stimulating hepatic mitochondrial oxidative activity and halting WAT lipolysis during refeeding (88). Loss of ERRα prevented the transcriptional repression of the mouse *Fgf21* gene during the transition from a fasted to fed state, which is consistent with the impaired clearance of fasting-induced NAFLD in the absence of ERRα (88). Conversely, ablation of ERRα exacerbated rapamycin-induced NAFLD (23). Rapamycin treatment of ERRα-null mice reduces the expression of TCA enzymes in liver and enhances mRNA levels of genes involved in lipogenesis including *Acly, Fasn*, and *Scd1*. Consequently, citrate accumulates and is shuttled into the lipogenic pathway, promoting hepatic TG accumulation.

### ERRα Transcriptional Regulation of the Mitochondrial TCA Cycle and Electron Transport Chain

If the liver is central to energy homeostasis at the body level, mitochondria are the metabolic hubs at the cellular level. Consistent with Figure 1, the GO cellular component analysis of hepatic ERRα target genes identified “mitochondria” as the top term, demonstrating that a primary function of ERRα is to regulate hepatic mitochondrial activity. It has been shown that overexpression of ERRα or PGC-1α enhances respiration capacity via promoting mitochondrial biogenesis and activity. On the other hand, loss of ERRα function leads to mitochondrial dysfunction and impaired ATP production partly by compromising the ability of PGC-1α to increase mitochondrial DNA content and to induce the expression of genes encoding mitochondrial proteins (20, 34, 39, 102, 113, 117–119). Functional genomic studies have identified ERRα as a comprehensive and genuine master regulator of the nuclear-encoded mitochondrial transcriptome. ERRα exerts its regulatory function via occupying the promoter regions of more than 700 genes encoding mitochondrial proteins, which are involved in all aspects of mitochondrial biogenesis and function (11, 120). No other transcription factor has been shown to control mitochondrial physiology and function as extensively. ChIP-seq analysis of ERRα binding in mouse liver showed that it binds to the regulatory regions of most genes encoding the enzymes involved in the TCA cycle, including: *Aco2, Idh3a, Idh3b, Sdha, Sdhb, Sdhc, Sdhd, Ogdh, Cs*, and *Fh1* (23). ERRα also occupies regulatory regions in the proximity of more than one hundred genes involved in the mitochondrial electron transport chain (ETC), including members of the NADH dehydrogenase complex, ubiquinol-cytochrome c reductases, several cytochrome c oxidase subunits, and the ATPase superfamily (23). Overall, as a master regulator of mitochondrial activity, ERRα positively regulates oxidative gene expression, aerobic respiration and ATP synthesis.

### ERRα Transcriptional Regulation of Liver Functions Beyond Metabolism

The liver has an architectural organization in which hepatocytes are in close proximity to immune cells and have immediate access to a vast network of blood vessels, enabling continuous and dynamic interactions between immune and metabolic responses (12, 121). In addition to its role in regulating energy metabolism, ERRα is also important in pathogen resistance and cancer development (25, 42, 44). Remarkably, ERRα plays a role in inflammation-related hepatocellular carcinoma (HCC) development. Global loss of ERRα activity promotes HCC following administration of the chemical carcinogen diethylnitrosamine (DEN) (101). Due to a deficiency in energy production, ERRα-null mice mediate DEN-induced cell death primarily by necrosis as opposed to apoptosis, an ATP-consuming process. In addition, the ERRα ChIP-seq study in mouse liver revealed that the gene *Nfkbia*, encoding the NF-κB suppressor IκBα, is a direct transcriptional target of ERRα. Further experiments confirmed that ERRα positively regulates IκBα expression in both hepatocytes and Kupffer cells. Thus, loss of ERRα resulted in enhanced NF-κB activity and subsequent cytokine gene activation in Kupffer cells, driving compensatory hepatocyte proliferation and HCC in response to DEN (101).

## ERRα in Liver Health and Disease

**Figure 3:**
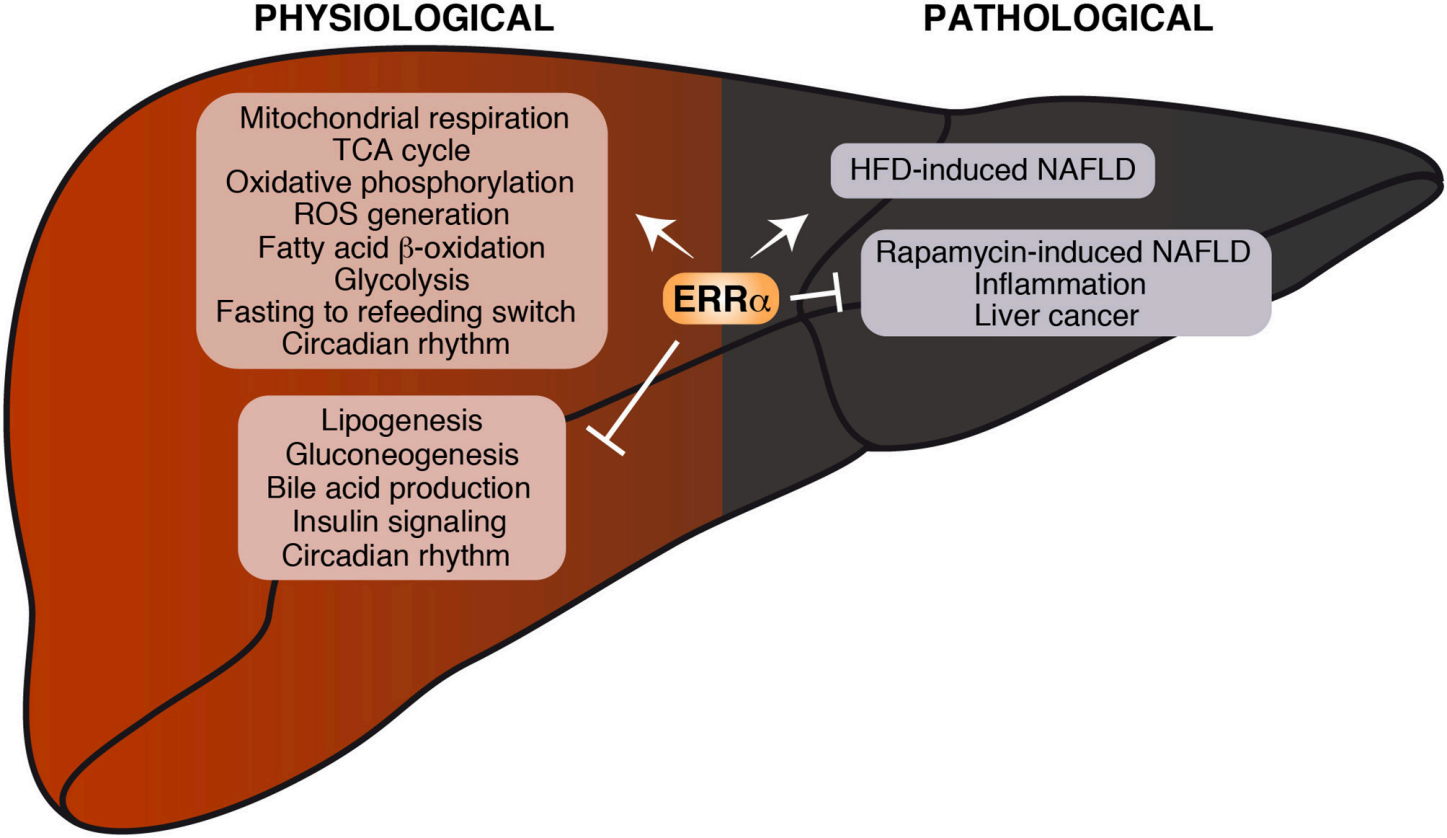
Current knowledge on the role of ERRα in liver physiology and pathology. Biological processes found either positively or negatively regulated by ERRα are shown. Under physiological conditions, ERRα can stimulate mitochondrial function and nutrient catabolism and repress *de novo* lipogenesis, gluconeogenesis and insulin signaling. Under pathological conditions, ERRα promotes the development of HFD-induced NAFLD on one hand and represses inflammation and protects from the development of rapamycin-induced NAFLD and liver cancer on the other.

Due to the complex network of coregulators and overlapping pathways as well as compensatory signaling mechanisms, the extent to which ERRα exerts its regulatory control *in vivo* may be difficult to predict. Although some of the anticipated effects and the consequent metabolic diseases caused by ERRα dysregulation may not necessarily be phenotypically manifested in the whole animal, ERRα clearly plays a central role in liver under both physiologic and pathologic conditions (Figure 3).

The liver plays a crucial role in regulating whole-body energy metabolism via crosstalk with other tissues including skeletal muscle, adipose, gut, and the brain in response to different environmental cues such as the switch from fasting to feeding, acting as a hub to metabolically connect various tissues. Dysfunction of liver signal transduction and nutrient metabolism contributes to the progression of insulin resistance, type 2 diabetes and NAFLD (12). NAFLD can progress from hepatic steatosis to non-alcoholic steatohepatitis (NASH), and can further progress to a more severe state of liver cirrhosis and HCC (122).

### ERRα in Insulin-Resistance and Diabetes

Insulin resistance, the condition in which a cell, tissue, or organism fails to respond appropriately to insulin, is a hallmark for the development of type 2 diabetes and a major contributor to the pathogenesis of NAFLD (12, 104). Obesity which is associated with chronic inflammation can also lead to insulin resistance (121). Liver, muscle, and adipose tissue are the organs most responsible for insulin-dependent glucose production and disposal. Insulin promotes glucose uptake into tissues such as muscle and adipose and simultaneously represses glucose production in the liver. Mitochondria, as the cellular powerhouse, is tightly associated with insulin sensitivity (123). A large body of data converges to support that transcriptional regulation plays a major role in the development of insulin resistance (13, 104). Previous findings have demonstrated reduced levels of ERRα-regulated genes in insulin-resistant humans (34), as well as the correlation between insulin sensitivity and ERRα mRNA expression in human adipose tissue (124). Furthermore, ERRα function has been shown to contribute to the development of insulin resistance in human diabetic muscle via down-regulating OXPHOS genes (48, 125), indicating the beneficial effects of enhancing ERRα activity in skeletal muscle on glucose uptake and handling. By contrast, in livers of patients with type 2 diabetes, OXPHOS genes are up-regulated and positively correlate with ERRα mRNA (126). Accordingly, hepatic insulin resistance is associated with increased mitochondrial respiration (127, 128). It has been proposed that in the context of hepatic insulin resistance, hyperinsulinemia increases hepatic lipogenesis and exacerbates fatty liver, in turn further increasing insulin resistance (13). Here we suggest that this vicious cycle can be reversed through antagonizing ERRα activity given that inhibition of ERRα decreases blood insulin levels, increases insulin sensitivity and protects animals from HFD-induced fatty liver (88, 100, 114). Together, there is compelling evidence to support a major role of ERRα as a transcriptional regulator of insulin action and changes in ERRα expression, DNA binding, PTMs, and cofactors recruitment could be linked to pathological changes in insulin resistance and diabetes through altering the expression of ERRα target genes.

## ERRα in NAFLD

Exogenous lipids (diet), *de novo* lipogenesis and adipose tissue lipolysis are the three main sources of hepatic fatty acids (FAs). Excess accumulation of triglycerides in hepatocytes leads to NAFLD, the most common chronic liver disorder in Western countries. Many theories have been proposed for the pathogenesis of NAFLD, including the “two-hits,” “multi-hits,” and “distinct-hits” models. In common, these hypotheses suggest that insulin resistance and oxidative stress are associated with NAFLD (129). Remarkably, ERRα contributes to the development of NAFLD in a context-dependent manner. On one hand, absence of ERRα impairs the development of NAFLD in response to increased dietary fat intake (88). While the expression of ERRα- and PGC-1α-encoding genes are upregulated in WT mice under HFD, this response is likely an adaptive response to mitochondrial dysfunction (130). In light that ERRα deficiency increases the susceptibility of mice to rapamycin-induced NAFLD (23) and impairs the reversal of fasting-induced NAFLD during refeeding (88), inducing ERRα activity appears more beneficial to treat and reverse the instilled disease.

### ERRα in Hepatocellular Carcinoma

Chronic inflammation associated with NAFLD, together with obesity and mitochondrial reactive oxygen species (ROS) accumulation promote HCC development, the terminal stage of liver disease (129). During tumorigenesis, cells undergo a metabolic switch from mitochondrial OXPHOS to glycolysis, a phenomenon referred to as the Warburg effect (131). ERRα has been shown to influence tumorigenesis via substrate utilization, modulation of metabolic pathways and transcriptional regulation of key oncogenes (9, 24, 25, 45, 132). ERRα also contributes to ROS production and detoxification (25, 42, 101). Although the precise role of ERRα in the progression from NAFLD to HCC is unclear, loss of ERRα promotes carcinogen-induced liver cancer in mice despite the lower ROS levels observed due to the de-repression of an NF-κB-mediated inflammatory response (101).

## Conclusions

Research findings presented in this review underscore a prominent role of ERRα in the regulation of hepatic homeostasis via direct modulation of an extensive range of metabolically-relevant genes and programs. Alongside a continued search for the natural endogenous ligand of ERRα, a key goal for the future is to exploit pharmaceuticals to modulate the transcriptional activity of ERRα to prevent and treat human diseases related to metabolic dysfunction. Several synthetic molecules have been shown to inhibit the constitutive transcriptional activity of ERRα including compound A (133), XCT790, compound 29 (C29), and compound 50 (C50) that act as ERRα inverse agonists (100, 114, 134). Consistent with the results obtained using ERRα-null mice as a model, treatment with the inverse agonist C29 led to normalized insulin and circulating triglyceride levels, improved insulin sensitivity and glucose tolerance in diet-induced obesity (DIO) mouse models as well as an overt diabetic rat model (100). Chronic administration of C50 showed similar beneficial effects in two murine models of obesity and insulin resistance (114). Interestingly, C29 and C50 modulate ERRα's activity in a tissue-specific manner. Given the beneficial effects of ERRα in skeletal muscle, inhibition of ERRα in the liver or adipose tissue, accompanied by its activation in skeletal muscle would be a therapeutic avenue for the treatment of the metabolic syndrome. As discussed above, ERRα is subject to the regulation by PTMs, which affect ERRα protein stability or physical interaction with coregulators. Future drug discovery relating to the specific regulation of ERRα transcriptional activity through PTMs would require further exploration of the physiologic, nutrient and hormonal cues that lead to the specific PTMs of ERRα and their relevant effects on cellular metabolism. Known compounds such as kinase inhibitors may exert their beneficial effects in the treatment of the metabolic syndrome via alteration of ERRα transcriptional activity either directly or indirectly. It is reasonable to hypothesize that combination therapies might lead to the desired beneficial anti-diabetic outcome but with less unwanted adverse effects. While it is clear that targeting ERRα activity in the liver may have therapeutic potential, future research and drug development will have to take into account the roles played by the three ERRs in the complex interplay between all metabolic tissues in the development of the metabolic syndrome and associated ailments.

## Author Contributions

HX, CD and VG participated in the design and writing of the review and have approved it for publication. CD analyzed data and generated the figures.

### Conflict of Interest Statement

The authors declare that the research was conducted in the absence of any commercial or financial relationships that could be construed as a potential conflict of interest.

## Supplementary Material

Table S1 List of ERRα-targeted genes with evidence for its regulation of these genes from ERRα activity perturbation studies in hepatocytes.

## References

[1] Evans RM Mangelsdorf DJ. Nuclear Receptors, RXR, and the Big Bang. Cell. (2014) 157:255–66. 10.1016/j.cell.2014.03.012 24679540 PMC4029515

[2] Evans RM. The nuclear receptor superfamily: a rosetta stone for physiology. Mol Endocrinol. (2005) 19:1429–38. 10.1210/me.2005-0046 15914712

[3] Giguère V. Orphan nuclear receptors: from gene to function. Endocr Rev. (1999) 20:689–725. 10.1210/er.20.5.689 10529899

[4] Mullican SE Dispirito JR Lazar MA. The orphan nuclear receptors at their 25-year reunion. J Mol Endocrinol. (2013) 51:T115–40. 10.1530/JME-13-0212 24096517 PMC3845022

[5] Giguère V Yang N Segui P Evans RM. Identification of a new class of steroid hormone receptors. Nature. (1988) 331:91–94. 10.1038/331091a0 3267207

[6] Giguère V. To ERR in the estrogen pathway. Trends Endocrinol Metab. (2002) 13:220–225. 10.1016/S1043-2760(02)00592-1 12185669

[7] Giguère V. Transcriptional control of energy homeostasis by the estrogen-related receptors. Endocr Rev. (2008) 29:677–696. 10.1210/er.2008-0017 18664618

[8] Audet-Walsh E Giguère V. The multiple universes of estrogen-related receptor α and γ in metabolic control and related diseases. Acta pharmacologica Sinica. (2015) 36:51–61. 10.1038/aps.2014.121 25500872 PMC4571319

[9] Deblois G Giguère V. Oestrogen-related receptors in breast cancer: control of cellular metabolism and beyond. Nat Rev Cancer. (2013) 13:27–36. 10.1038/nrc3396 23192231

[10] Villena JA Kralli A. ERRα: a metabolic function for the oldest orphan. Trends Endocrinol Metab. (2008) 19:269–76. 10.1016/j.tem.2008.07.005 18778951 PMC2786240

[11] Eichner LJ Giguère V. Estrogen-related receptors (ERRs): a new dawn in the control of mitochondrial gene networks. Mitochondrion. (2011) 11:544–552. 10.1016/j.mito.2011.03.121 21497207

[12] Rui L. Energy metabolism in the liver. Compr Physiol. (2014) 4:177–97. 10.1002/cphy.c130024 24692138 PMC4050641

[13] Moore DD. Nuclear receptors reverse McGarry's vicious cycle to insulin resistance. Cell Metab. (2012) 15:615–22. 10.1016/j.cmet.2012.03.016 22560214 PMC3613429

[14] Lu NZ Wardell SE Burnstein KL Defranco D Fuller PJ Giguere V. International Union of Pharmacology. LXV. The pharmacology and classification of the nuclear receptor superfamily: glucocorticoid, mineralocorticoid, progesterone, and androgen receptors. Pharmacol Rev. (2006) 58:782–97. 10.1124/pr.58.4.9 17132855

[15] Tremblay AM Giguère V. The NR3B subgroup: an ovERRview. Nucl Recept Signal. (2007) 5:e009. 10.1621/nrs.05009 18174917 PMC2121319

[16] Eudy JD Yao S Weston MD Ma-Edmonds M Talmage CB Cheng JJ. Isolation of a gene encoding a novel member of the nuclear receptor superfamily from the critical region of Usher syndrome type IIa at 1q41. Genomics. (1998) 50:382–4. 10.1006/geno.1998.5345 9676434

[17] Tremblay AM Wilson BJ Yang XJ Giguère V Phosphorylation-dependent sumoylation regulates ERRα and γ transcriptional activity through a synergy control motif. Mol Endocrinol. (2008) 22:570–84. 10.1210/me.2007-0357 18063693 PMC5419619

[18] Vu EH Kraus RJ Mertz JE. Phosphorylation-dependent sumoylation of estrogen-related receptor α1. Biochemistry. (2007) 46:9795–804. 10.1021/bi700316g 17676930

[19] Sladek R Bader J-A Giguère V. The orphan nuclear receptor estrogen-related receptor α is a transcriptional regulator of the human medium-chain acyl coenzyme A dehydrogenase gene. Mol Cell Biol. (1997) 17:5400–5409. 10.1128/MCB.17.9.5400 9271417 PMC232390

[20] Dufour CR Wilson BJ Huss JM Kelly DP Alaynick WA Downes M Genome-wide orchestration of cardiac functions by orphan nucler receptors ERRα and γ. Cell Metab. (2007) 5:345–56. 10.1016/j.cmet.2007.03.007 17488637

[21] Deblois G Hall JA Perry MC Laganière J Ghahremani M Park M. Genome-wide identification of direct target genes implicates estrogen-related receptor α as a determinant of breast cancer heterogeneity. Cancer Res. (2009) 69:6149–57. 10.1158/0008-5472.CAN-09-1251 19622763

[22] Charest-Marcotte A Dufour CR Wilson BJ Tremblay AM Eichner LJ Arlow DH The homeobox protein Prox1 is a negative modulator of ERRα/PGC-1α bioenergetic functions. Genes Dev. (2010) 24:537–42. 10.1101/gad.1871610 20194433 PMC2841331

[23] Chaveroux C Eichner LJ Dufour CR Shatnawi A Khoutorsky A Bourque G Molecular and genetic crosstalks between mTOR and ERRα are key determinants of rapamycin-induced non-alcoholic fatty liver. Cell Metab. (2013) 17:586–98. 10.1016/j.cmet.2013.03.003 23562079

[24] Audet-Walsh E Papadopoli DJ Gravel SP Yee T Bridon G Caron M. The PGC-1α/ERRα axis represses one-carbon metabolism and promotes sensitivity to anti-folate therapy in breast cancer. Cell Rep. (2016) 14:920–31. 10.1016/j.celrep.2015.12.086 26804918

[25] Deblois G Smith HW Tam IS Gravel SP Caron M Savage P ERR? mediates metabolic adaptations driving lapatinib resistance in breast cancer. Nat Commun. (2016) 7:12156 10.1038/ncomms12156 27402251 PMC4945959

[26] Chen X Xu H Yuan P Fang F Huss M Vega VB. Integration of external signaling pathways with the core transcriptional network in embryonic stem cells. Cell. (2008) 133:1106–17. 10.1016/j.cell.2008.04.043 18555785

[27] Gearhart MD Holmbeck SM Evans RM Dyson HJ Wright PE. Monomeric complex of human orphan estrogen related receptor-2 with DNA: a pseudo-dimer interface mediates extended half-site recognition. J Mol Biol. (2003) 327:819–32. 10.1016/S0022-2836(03)00183-9 12654265

[28] Barry JB Laganière J Giguère V A single nucleotide in an estrogen related receptor α site can dictate mode of binding and PGC-1α activation of target promoters. Mol Endocrinol. (2006) 20:302–10. 10.1210/me.2005-0313 16150865

[29] Huppunen J Aarnisalo P. Dimerization modulates the activity of the orphan nuclear receptor ERRγ. Biochem Biophys Res Commun. (2004) 314:964–70. 10.1016/j.bbrc.2003.12.194 14751226

[30] Greschik H Wurtz JM Sanglier S Bourguet W van Dirsselaer A Moras D. Structural and functional evidence for ligand-independent transcriptional activation by the estrogen-related receptor 3. Mol Cell. (2002) 9:303–13. 10.1016/S1097-2765(02)00444-6 11864604

[31] Kallen J Schlaeppi JM Bitsch F Filipuzzi I Schilb A Riou V. Evidence for ligand-independent transcriptional activation of the human estrogen-related receptor α (ERRα): crystal structure of ERRα ligand binding domain in complex with peroxisome proliferator-activated receptor coactivator-1α. J Biol Chem. (2004) 279:49330–7. 10.1074/jbc.M407999200 15337744

[32] Kamei Y Ohizumi H Fujitani Y Nemoto T Tanaka T Takahashi N. PPARγ coactivator 1β/ERR ligand 1 is an ERR protein ligand, whose expression induces a high-energy expenditure and antagonizes obesity. Proc Natl Acad Sci USA. (2003) 100:12378–83. 10.1073/pnas.2135217100 14530391 PMC218766

[33] Huss JM Kopp RP Kelly DP. Peroxisome proliferator-activated receptor coactivator-1α (PGC-1α) coactivates the cardiac-enriched nuclear receptors estrogen-related receptor-α and -γ. Identification of novel leucine-rich interaction motif within PGC-1α. J Biol Chem. (2002) 277:40265–74. 10.1074/jbc.M206324200 12181319

[34] Schreiber SN Emter R Hock MB Knutti D Cardenas J Podvinec M. The estrogen-related receptor alpha (ERRα) functions in PPARγ coactivator 1α (PGC-1α)-induced mitochondrial biogenesis. Proc Natl Acad Sci USA. (2004) 101:6472–7. 10.1073/pnas.0308686101 15087503 PMC404069

[35] Schreiber SN Knutti D Brogli K Uhlmann T Kralli A. The transcriptional coactivator PGC-1 regulates the expression and activity of the orphan nuclear receptor ERRα. J Biol Chem. (2003) 278:9013–8. 10.1074/jbc.M212923200 12522104

[36] Laganière J Tremblay GB Dufour CR Giroux S Rousseau F Giguère V A polymorphic autoregulatory hormone response element in the human estrogen related receptor α (ERRα) promoter dictates PGC-1α control of ERRα expression. J Biol Chem. (2004) 279:18504–10. 10.1074/jbc.M313543200 14978033

[37] Wende AR Huss JM Schaeffer PJ Giguère V Kelly DP. PGC-1α coactivates PDK4 gene expression via the orphan nuclear receptor ERRα: a mechanism for transcriptional control of muscle glucose metabolism. Mol Cell Biol. (2005) 25:10684–94. 10.1128/MCB.25.24.10684-10694.2005 16314495 PMC1316952

[38] Villena JA. New insights into PGC-1 coactivators: redefining their role in the regulation of mitochondrial function and beyond. Febs J. (2015) 282:647–72. 10.1111/febs.13175 25495651

[39] Finck BN Kelly DP. PGC-1 coactivators: inducible regulators of energy metabolism in health and disease. J Clin Invest. (2006) 116:615–22. 10.1172/JCI27794 16511594 PMC1386111

[40] Vega RB Kelly DP. A role for estrogen-related receptor α in the control of mitochondrial fatty acid β-oxidation during brown adipocyte differentiation. J Biol Chem. (1997) 272:31693–9. 10.1074/jbc.272.50.31693 9395511

[41] Puigserver P Wu Z Park CW Graves R Wright M Spiegelman BM. A cold-inducible coactivator of nuclear receptors linked to adaptive thermogenesis. Cell. (1998) 92:829–39. 10.1016/S0092-8674(00)81410-5 9529258

[42] Sonoda J Laganière J Mehl IR Barish GD Chong LW Li X Nuclear receptor ERRα and coactivator PGC-1β are effectors of IFN-γ induced host defense. Genes Dev. (2007) 21:1909–20. 10.1101/gad.1553007 17671090 PMC1935029

[43] Sonoda J Mehl IR Chong LW Nofsinger RR Evans RM. PGC-1β controls mitochondrial metabolism to modulate circadian activity, adaptive thermogenesis, and hepatic steatosis. Proc Natl Acad Sci USA. (2007) 104:5223–8. 10.1073/pnas.0611623104 17360356 PMC1829290

[44] Deblois G St-Pierre J Giguère V. The PGC-1/ERR signaling axis in cancer. Oncogene. (2013) 32:3483–90. 10.1038/onc.2012.529 23208510

[45] Deblois G Chahrour G Perry MC Sylvain-Drolet G Muller WJ Giguère V. Transcriptional control of the ERBB2 amplicon by ERRα and PGC-1β promotes mammary gland tumorigenesis. Cancer Res. (2010) 70:10277–87. 10.1158/0008-5472.CAN-10-2840 20961995

[46] Shao D Liu Y Liu X Zhu L Cui Y Cui A PGC-1β-regulated mitochondrial biogenesis and function in myotubes is mediated by NRF-1 and ERRα. Mitochondrion. (2010) 10:516–27. 10.1016/j.mito.2010.05.012 20561910

[47] Emmett MJ Lim HW Jager J Richter HJ Adlanmerini M Peed LC. Histone deacetylase 3 prepares brown adipose tissue for acute thermogenic challenge. Nature. (2017) 546:544–8. 10.1038/nature22819 28614293 PMC5826652

[48] Mootha VK Handschin C Arlow D Xie X St Pierre J Sihag S ERRα and GABPAα/β specify PGC-1α-dependent oxidative phosphorylation gene expression that is altered in diabetic muscle. Proc Natl Acad Sci USA. (2004) 101:6570–5. 10.1073/pnas.0401401101 15100410 PMC404086

[49] Gaillard S Dwyer MA McDonnell DP. Definition of the molecular basis for estrogen receptor-related receptor-α-cofactor interactions. Mol Endocrinol. (2007) 21:62–76. 10.1210/me.2006-0179 17053040

[50] Mottis A Mouchiroud L Auwerx J. Emerging roles of the corepressors NCoR1 and SMRT in homeostasis. Genes Dev. (2013) 27:819–35. 10.1101/gad.214023.113 23630073 PMC3650221

[51] Perez-Schindler J Summermatter S Salatino S Zorzato F Beer M Balwierz PJ. The corepressor NCoR1 antagonizes PGC-1alpha and estrogen-related receptor alpha in the regulation of skeletal muscle function and oxidative metabolism. Mol Cell Biol. (2012) 32:4913–24. 10.1128/MCB.00877-12 23028049 PMC3510532

[52] Yamamoto H Williams EG Mouchiroud L Canto C Fan W Downes M. NCoR1 is a conserved physiological modulator of muscle mass and oxidative function. Cell. (2011) 147:827–39. 10.1016/j.cell.2011.10.017 22078881 PMC3225739

[53] Rosenfeld MG Lunyak VV Glass CK. Sensors and signals: a coactivator/corepressor/epigenetic code for integrating signal-dependent programs of transcriptional response. Genes Dev. (2006) 20:1405–28. 10.1101/gad.1424806 16751179

[54] Powelka AM Seth A Virbasius JV Kiskinis E Nicoloro SM Guilherme A. Suppression of oxidative metabolism and mitochondrial biogenesis by the transcriptional corepressor RIP140 in mouse adipocytes. J Clin Invest. (2006) 116:125–36. 10.1172/JCI26040 16374519 PMC1319222

[55] Castet A Herledan A Bonnet S Jalaguier S Vanacker JM Cavailles V. Receptor-interacting protein 140 differentially regulates estrogen receptor-related receptor transactivation depending on target genes. Mol Endocrinol. (2006) 20:1035–47. 10.1210/me.2005-0227 16439465

[56] Debevec D Christian M Morganstein D Seth A Herzog B Parker MG. Receptor interacting protein 140 regulates expression of uncoupling protein 1 in adipocytes through specific peroxisome proliferator activated receptor isoforms and estrogen-related receptor α. Mol Endocrinol. (2007) 21:1581–92. 10.1210/me.2007-0103 17456798 PMC2072047

[57] Christian M Kiskinis E Debevec D Leonardsson G White R Parker MG. RIP140-targeted repression of gene expression in adipocytes. Mol Cell Biol. (2005) 25:9383–91. 10.1128/MCB.25.21.9383-9391.2005 16227589 PMC1265803

[58] Christian M White R Parker MG. Metabolic regulation by the nuclear receptor corepressor RIP140. Trends Endocrinol Metab. (2006) 17:243–50. 10.1016/j.tem.2006.06.008 16815031

[59] Nichol D Christian M Steel JH White R Parker MG. RIP140 expression is stimulated by estrogen-related receptor alpha during adipogenesis. J Biol Chem. (2006) 281:32140–7. 10.1074/jbc.M604803200 16923809

[60] Treiber T Treiber N Meister G. Regulation of microRNA biogenesis and its crosstalk with other cellular pathways. Nat Rev Mol Cell Biol. (2018) 20:5–20. 10.1038/s41580-018-0059-1 30728477

[61] Gebert LFR MacRae IJ. Regulation of microRNA function in animals. Nat Rev Mol Cell Biol. (2018) 20:21–37. 10.1038/s41580-018-0045-7 30108335 PMC6546304

[62] Zhao Y Li Y Lou G Zhao L Xu Z Zhang Y. MiR-137 targets estrogen-related receptor α and impairs the proliferative and migratory capacity of breast cancer cells. PLoS ONE. (2012) 7:e39102. 10.1371/journal.pone.0039102 22723937 PMC3377602

[63] Lu TM Lu W Zhao LJ. MicroRNA-137 affects proliferation and migration of placenta trophoblast cells in preeclampsia by targeting ERRalpha. Reprod Sci. (2017) 24:85–96. 10.1177/1933719116650754 27270272

[64] Tiwari A Shivananda S Gopinath KS Kumar A. MicroRNA-125a reduces proliferation and invasion of oral squamous cell carcinoma cells by targeting estrogen-related receptor α: implications for cancer therapeutics. J Biol Chem. (2014) 289:32276–90. 10.1074/jbc.M114.584136 25266720 PMC4231701

[65] Ji HL Song CC Li YF He JJ Li YL Zheng XL. miR-125a inhibits porcine preadipocytes differentiation by targeting ERRalpha. Mol Cell Biochem. (2014) 395:155–65. 10.1007/s11010-014-2121-4 24952481

[66] Ren Y Jiang H Ma D Nakaso K Feng J. Parkin degrades estrogen-related receptors to limit the expression of monoamine oxidases. Hum Mol Genet. (2011) 20:1074–83. 10.1093/hmg/ddq550 21177257 PMC3043659

[67] Barry JB Giguère V. Epidermal growth factor-induced signaling in breast cancer cells results in selective target gene activation by orphan nuclear receptor estrogen-related receptor α. Cancer Res. (2005) 65:6120–9. 10.1158/0008-5472.CAN-05-0922 16024613

[68] Ariazi EA Kraus RJ Farrell ML Jordan VC Mertz JE. Estrogen-related receptor α1 transcriptional activities are regulated in part via the ErbB2/HER2 signaling pathway. Mol Cancer Res. (2007) 5:71–85. 10.1158/1541-7786.MCR-06-0227 17259347

[69] Liu D Benlhabib H Mendelson CR. cAMP enhances estrogen-related receptor alpha (ERRalpha) transcriptional activity at the SP-A promoter by increasing its interaction with protein kinase A and steroid receptor coactivator 2 (SRC-2). Mol Endocrinol. (2009) 23:772–83. 10.1210/me.2008-0282 19264843 PMC2691680

[70] Ning Z Du X Zhang J Yang K Miao L Zhu Y. PGE2 modulates the transcriptional activity of ERRa in prostate stromal cells. Endocrine. (2014) 47:901–12. 10.1007/s12020-014-0261-7 24760659

[71] Wilson BJ Tremblay AM Deblois G Sylvain-Drolet G Giguère V. An acetylation switch modulates the transcriptional activity of estrogen-related recetpor α. Mol Endocrinol. (2010) 24:1349–58. 10.1210/me.2009-0441 20484414 PMC5417470

[72] Jo YS Ryu D Maida A Wang X Evans RM Schoonjans K. Phosphorylation of the nuclear receptor corepressor 1 by protein kinase B switches its corepressor targets in the liver in mice. Hepatology. (2015) 62:1606–18. 10.1002/hep.27907 25998209 PMC4618256

[73] Vianna CR Huntgeburth M Coppari R Choi CS Lin J Krauss S. Hypomorphic mutation of PGC-1β causes mitochondrial dysfunction and liver insulin resistance. Cell Metab. (2006) 4:453–64. 10.1016/j.cmet.2006.11.003 17141629 PMC1764615

[74] Zhang Z Teng CT Estrogen receptor-related receptor α1 interacts with coactivator and constitutively activates the estrogen response elements of the human lactoferrin gene. J Biol Chem. (2000) 275:20837–46. 10.1074/jbc.M001880200 10779508

[75] Xie W Hong H Yang NN Lin RJ Simon CM Stallcup MR. Constitutive activation of transcription and binding of coactivator by estrogen-related receptors 1 and 2. Mol Endocrinol. (1999) 13:2151–62. 10.1210/mend.13.12.0381 10598588

[76] Zhou D Chen S. PNRC2 is a 16 kDa coactivator that interacts with nuclear receptors through an SH3-binding motif. Nucleic Acids Res. (2001) 29:3939–48. 10.1093/nar/29.19.3939 11574675 PMC60244

[77] Villena JA Hock MB Giguère V Kralli A Orphan nuclear receptor ERRα is essential for adaptive thermogenesis. Proc Natl Acad Sci USA. (2007) 104:1418–23. 10.1073/pnas.0607696104 17229846 PMC1783094

[78] Brown EL Hazen BC Eury E Wattez JS Gantner ML Albert V. Estrogen-related receptors mediate the adaptive response of brown adipose tissue to adrenergic stimulation. iScience. (2018) 2:221–237. 10.1016/j.isci.2018.03.005 29888756 PMC5993202

[79] Gidlund EK Ydfors M Appel S Rundqvist H Sundberg CJ Norrbom J. Rapidly elevated levels of PGC-1alpha-b protein in human skeletal muscle after exercise: exploring regulatory factors in a randomized controlled trial. J Appl Physiol. (2015) 119:374–84. 10.1152/japplphysiol.01000.2014 26089547

[80] Reitzner SM Norrbom J Sundberg CJ Gidlund EK. Expression of striated activator of rho-signaling in human skeletal muscle following acute exercise and long-term training. Physiol Rep. (2018) 6:e13624. 10.14814/phy2.13624 29504288 PMC5835521

[81] Cartoni R Leger B Hock MB Praz M Crettenand A Pich S. Mitofusins 1/2 and ERRα expression are increased in human skeletal muscle after physical exercise. J Physiol. (2005) 567:349–58. 10.1113/jphysiol.2005.092031 15961417 PMC1474174

[82] Perry MC Dufour CR Tam IS B'Chir W Giguère V. Estrogen-related receptor-α coordinates transcriptional programs essential for exercise tolerance and muscle fitness. Mol Endocrinol. (2014) 28:2060–71. 10.1210/me.2014-1281 25361393 PMC5414781

[83] Tremblay AM Dufour CR Ghahremani M Reudelhuber TL Giguère V. Physiological genomics identifies estrogen-related receptor α as a regulator of renal sodium and potassium homeostasis and the renin-angiotensin pathway. Mol Endocrinol. (2010) 24:22–32. 10.1210/me.2009-0254 19901197 PMC5428150

[84] Dufour CR Levasseur M-P Pham NHH Eichner LJ Wilson BJ Charest-Marcotte A. Genomic convergence among ERRα, Prox1 and Bmal1 in the control of metabolic clock outputs. PLoS Genet. (2011) 7:e1002143. 10.1371/journal.pgen.1002143 21731503 PMC3121748

[85] Horard B Rayet B Triqueneaux G Laudet V Delaunay F Vanacker JM. Expression of the orphan nuclear receptor ERRα is under circadian regulation in estrogen-responsive tissues. J Mol Endocrinol. (2004) 33:87–97. 10.1677/jme.0.0330087 15291745

[86] Yang X Downes M Yu RT Bookout AL He W Straume M. Nuclear receptor expression links the circadian clock to metabolism. Cell. (2006) 126:801–10. 10.1016/j.cell.2006.06.050 16923398

[87] Kettner NM Voicu H Finegold MJ Coarfa C Sreekumar A Putluri N. Circadian homeostasis of liver metabolism suppresses hepatocarcinogenesis. Cancer Cell. (2016) 30:909–24. 10.1016/j.ccell.2016.10.007 27889186 PMC5695235

[88] B'Chir W Dufour CR Ouellet C Yan M Tam IS Andrzejewski S. Divergent role of estrogen-related receptor alpha in lipid- and fasting-induced hepatic steatosis in mice. Endocrinology. (2018) 159:2153–64. 10.1210/en.2018-00115 29635284

[89] Ranhotra HS. Up-regulation of orphan nuclear estrogen-related receptor α expression during long-term caloric restriction in mice. Mol Cell Biochem. (2009) 332:59–65. 10.1007/s11010-009-0174-6 19504233

[90] Herzog B Cardenas J Hall RK Villena JA Budge PJ Giguère V. Estrogen-related receptor α is a repressor of phosphoenolpyruvate carboxykinase gene transcription. J Biol Chem. (2006) 281:99–106. 10.1074/jbc.M509276200 16267049

[91] Carrier JC Deblois G Champigny C Levy E Giguère V. Estrogen related-receptor α (ERRα) is a transcriptional regulator of apolipoprotein A-IV and controls lipid handling in the intestine. J Biol Chem. (2004) 279:52052–58. 10.1074/jbc.M410337200 15466464

[92] Soriano FX Liesa M Bach D Chan DC Palacin M Zorzano A. Evidence for a mitochondrial regulatory pathway defined by peroxisome proliferator-activated receptor-γ coactivator-1 α, estrogen-related receptor-α, and mitofusin 2. Diabetes. (2006) 55:1783–91. 10.2337/db05-0509 16731843

[93] Rangwala SM Li X Lindsley L Wang X Shaughnessy S Daniels TG. Estrogen-related receptor α is essential for the expression of antioxidant protection genes and mitochondrial function. Biochem Biophys Res Commun. (2007) 357:231–6. 10.1016/j.bbrc.2007.03.126 17418099

[94] Deblois G Giguère V. Nuclear receptor location analyses in mammalian genomes: from gene regulation to regulatory networks. Mol Endocrinol. (2008) 22:1999–2011. 10.1210/me.2007-0546 18292239 PMC5419453

[95] Connaughton S Chowdhury F Attia RR Song S Zhang Y Elam MB. Regulation of pyruvate dehydrogenase kinase isoform 4 (PDK4) gene expression by glucocorticoids and insulin. Mol Cell Endocrinol. (2010) 315:159–67. 10.1016/j.mce.2009.08.011 19703515 PMC2815206

[96] Gaillard S Grasfeder LL Haeffele CL Lobenhofer EK Chu TM Wolfinger R. Receptor-selective coactivators as tools to define the biology of specific receptor-coactivator pairs. Mol Cell. (2006) 24:797–803. 10.1016/j.molcel.2006.10.012 17157261

[97] He X Ma S Tian Y Wei C Zhu Y Li F. ERRalpha negatively regulates type I interferon induction by inhibiting TBK1-IRF3 interaction. PLoS Pathog. (2017) 13:e1006347. 10.1371/journal.ppat.1006347 28591144 PMC5476288

[98] Grasfeder LL Gaillard S Hammes SR Ilkayeva O Newgard CB Hochberg RB. Fasting-induced hepatic production of DHEA is regulated by PGC-1alpha, ERRalpha, and HNF4alpha. Mol Endocrinol. (2009) 23:1171–82. 10.1210/me.2009-0024 19389810 PMC2718748

[99] Singh BK Sinha RA Tripathi M Mendoza A Ohba K Sy JAC. Thyroid hormone receptor and ERRalpha coordinately regulate mitochondrial fission, mitophagy, biogenesis, and function. Sci Signal. (2018) 11:eaam5855. 10.1126/scisignal.aam5855 29945885

[100] Patch RJ Searle LL Kim AJ De D Zhu X Askari HB. Identification of diaryl ether-based ligands for estrogen-related receptor α as potential antidiabetic agents. J Med Chem. (2011) 54:788–808. 10.1021/jm101063h 21218783

[101] Hong E-J Levasseur M-P Dufour CR Perry M-C Giguère V Loss of estrogen-related receptor α promotes hepatocellular carcinogenesis development via metabolic and inflammatory disturbances. Proc Natl Acad Sci USA. (2013) 110:17975–80. 10.1073/pnas.1315319110 24127579 PMC3816417

[102] Buler M Aatsinki SM Izzi V Uusimaa J Hakkola J. SIRT5 is under the control of PGC-1alpha and AMPK and is involved in regulation of mitochondrial energy metabolism. FASEB J. (2014) 28:3225–37. 10.1096/fj.13-245241 24687991

[103] Zhang Y Ma K Sadana P Chowdhury F Gaillard S Wang F. Estrogen-related receptors stimulate pyruvate dehydrogenase kinase isoform 4 gene expression. J Biol Chem. (2006) 281:39897–906. 10.1074/jbc.M608657200 17079227

[104] Kang S Tsai LT Rosen ED. Nuclear Mechanisms of Insulin Resistance. Trends Cell Biol. (2016) 26:341–51. 10.1016/j.tcb.2016.01.002 26822036 PMC4844850

[105] Ichida M Nemoto S Finkel T Identification of a specific molecular repressor of the peroxisome proliferator-activated receptor γ coactivator-1α (PGC-α). J Biol Chem. (2002) 277:50991–5. 10.1074/jbc.M210262200 12397057

[106] Zhang Z Teng CT Interplay between estrogen-related receptor a (ERRa) and g (ERRg) on the regulation of ERRalpha gene expression. Mol Cell Endocrinol. (2007) 264:128–41. 10.1016/j.mce.2006.11.002 17157980 PMC1808420

[107] Zhu LL Liu Y Cui AF Shao D Liang JC Liu XJ. PGC-1alpha coactivates estrogen-related receptor-alpha to induce the expression of glucokinase. Am J Physiol Endocrinol Metab. (2010) 298:E1210–8. 10.1152/ajpendo.00633.2009 20215575

[108] Massa ML Gagliardino JJ Francini F. Liver glucokinase: an overview on the regulatory mechanisms of its activity. IUBMB Life. (2011) 63:1–6. 10.1002/iub.411 21280170

[109] Araki M Motojima K. Identification of ERRα as a specific partner of PGC-1α for the activation of PDK4 gene expression in muscle. Febs J. (2006) 273:1669–80. 10.1111/j.1742-4658.2006.05183.x 16623704

[110] Bookout AL Jeong Y Downes M Yu RT Evans RM Mangelsdorf DJ. Anatomical profiling of nuclear receptor expression reveals a hierarchical transcriptional network. Cell. (2006) 126:789–99. 10.1016/j.cell.2006.06.049 16923397 PMC6211849

[111] Huss JM Kelly DP. Nuclear receptor signaling and cardiac energetics. Circ Res. (2004) 95:568–78. 10.1161/01.RES.0000141774.29937.e3 15375023

[112] Kim MS Shigenaga JK Moser AH Feingold KR Grunfeld C. Suppression of estrogen-related receptor α and medium-chain acyl-coenzyme A dehydrogenase in the acute-phase response. J Lipid Res. (2005) 46:2282–8. 10.1194/jlr.M500217-JLR200 16061943

[113] Luo J Sladek R Carrier J Bader J-A Richard D Giguère V. Reduced fat mass in mice lacking orphan nuclear receptor estrogen-related receptor α. Mol Cell Biol. (2003) 23:7947–56. 10.1128/MCB.23.22.7947-7956.2003 14585956 PMC262360

[114] Patch RJ Huang H Patel S Cheung W Xu G Zhao BP. Indazole-based ligands for estrogen-related receptor alpha as potential anti-diabetic agents. Eur J Med Chem.(2017) 138:830–53. 10.1016/j.ejmech.2017.07.015 28735214

[115] Ju D He J Zhao L Zheng X Yang G. Estrogen related receptor alpha-induced adipogenesis is PGC-1beta-dependent. Mol Biol Rep. (2012) 39:3343–54. 10.1007/s11033-011-1104-8 21732060

[116] Ijichi N Ikeda K Horie-Inoue K Yagi K Okazaki Y Inoue S. Estrogen-related receptor alpha modulates the expression of adipogenesis-related genes during adipocyte differentiation. Biochem Biophys Res Commun. (2007) 358:813–8. 10.1016/j.bbrc.2007.04.209 17512501

[117] Willy PJ Murray IR Qian J Busch BB Stevens WC Jr Martin R. Regulation of PPARγ coactivator 1α (PGC-1α) signaling by an estrogen-related receptor α (ERRα) ligand. Proc Natl Acad Sci USA. (2004) 101:8912–8917. 10.1073/pnas.0401420101 15184675 PMC428446

[118] Huss JM Pineda Torra I Staels B Giguère V Kelly DP. Estrogen-related receptor α directs peroxisome proliferator-activated receptor α signaling in the transcriptional control of energy metabolism in cardiac and skeletal muscle. Mol Cell Biol. (2004) 24:9079–91. 10.1128/MCB.24.20.9079-9091.2004 15456881 PMC517878

[119] Lin J Handschin C Spiegelman BM. Metabolic control through the PGC-1 family of transcription coactivators. Cell Metab. (2005) 1:361–70. 10.1016/j.cmet.2005.05.004 16054085

[120] Deblois G Giguère V. Functional and physiological genomics of estrogen-related receptors (ERRs) in health and disease. Biochim Biophys Acta. (2011) 1812:1032–40. 10.1016/j.bbadis.2010.12.009 21172432

[121] Hotamisligil GS. Inflammation and metabolic disorders. Nature. (2006) 444:860–7. 10.1038/nature05485 17167474

[122] Brunt EM Wong VW Nobili V Day CP Sookoian S Maher JJ Nonalcoholic fatty liver disease. Nat Rev Dis Primers. (2015) 1:15080 10.1038/nrdp.2015.80 27188459

[123] Lagouge M Argmann C Gerhart-Hines Z Meziane H Lerin C Daussin F. Resveratrol improves mitochondrial function and protects against metabolic disease by activating SIRT1 and PGC-1α. Cell. (2006) 127:1109–22. 10.1016/j.cell.2006.11.013 17112576

[124] Rutanen J Yaluri N Modi S Pihlajamaki J Vanttinen M Itkonen P. SIRT1 mRNA expression may be associated with energy expenditure and insulin sensitivity. Diabetes. (2010) 59:829–35. 10.2337/db09-1191 20107110 PMC2844830

[125] Mootha VK Bunkenborg J Olsen JV Hjerrild M Wisniewski JR Stahl E. Integrated analysis of protein composition, tissue diversity, and gene regulation in mouse mitochondria. Cell. (2003) 115:629–40. 10.1016/S0092-8674(03)00926-7 14651853

[126] Misu H Takamura T Matsuzawa N Shimizu A Ota T Sakurai M. Genes involved in oxidative phosphorylation are coordinately upregulated with fasting hyperglycaemia in livers of patients with type 2 diabetes. Diabetologia. (2007) 50:268–77. 10.1007/s00125-006-0489-8 17187250

[127] Franko A von Kleist-Retzow JC Neschen S Wu M Schommers P Bose M. Liver adapts mitochondrial function to insulin resistant and diabetic states in mice. J Hepatol. (2014) 60:816–23. 10.1016/j.jhep.2013.11.020 24291365

[128] Jelenik T Sequaris G Kaul K Ouwens DM Phielix E Kotzka J. Tissue-specific differences in the development of insulin resistance in a mouse model for type 1 diabetes. Diabetes. (2014) 63:3856–67. 10.2337/db13-1794 24917575

[129] Piccinin E Villani G Moschetta A. Metabolic aspects in NAFLD, NASH and hepatocellular carcinoma: the role of PGC1 coactivators. Nat Rev Gastroenterol Hepatol. (2018) 16:160–74. 10.1038/s41575-018-0089-3 30518830

[130] Garcia-Ruiz I Solis-Munoz P Fernandez-Moreira D Grau M Munoz-Yague T Solis-Herruzo JA. NADPH oxidase is implicated in the pathogenesis of oxidative phosphorylation dysfunction in mice fed a high-fat diet. Sci Rep. (2016) 6:23664. 10.1038/srep23664 27173483 PMC4866080

[131] Warburg O. On respiratory impairment in cancer cells. Science. (1956) 124:269–70. 13351639

[132] Chang CY Kazmin D Jasper JS Kunder R Zuercher WJ McDonnell DP. The metabolic regulator ERRα, a downstream target of HER2/IGF-1R, as a therapeutic target in breast cancer. Cancer Cell. (2011) 20:500–10. 10.1016/j.ccr.2011.08.023 22014575 PMC3199323

[133] Chisamore MJ Wilkinson HA Flores O Chen JD. Estrogen-related receptor-alpha antagonist inhibits both estrogen receptor-positive and estrogen receptor-negative breast tumor growth in mouse xenografts. Mol Cancer Ther. (2009) 8:672–81. 10.1158/1535-7163.MCT-08-1028 19276159

[134] Busch BB Stevens WC Jr Martin R Ordentlich P Zhou S Sapp DW. Identification of a selective inverse agonist for the orphan nuclear receptor estrogen-related receptor α. J Med Chem. (2004) 47:5593–5596. 10.1021/jm049334f 15509154

